# Anxiety and Depressive Symptoms as Predictors of All-Cause Mortality among People with Insulin-Naïve Type 2 Diabetes: 17-Year Follow-Up of the Second Nord-Trøndelag Health Survey (HUNT2), Norway

**DOI:** 10.1371/journal.pone.0160861

**Published:** 2016-08-18

**Authors:** Marjolein M. Iversen, Giesje Nefs, Grethe S. Tell, Birgitte Espehaug, Kristian Midthjell, Marit Graue, Frans Pouwer

**Affiliations:** 1 Department of Health and Social Sciences, Centre for Evidence-Based Practice, Bergen University College, Bergen, Norway; 2 Department of Medicine, Section of Endocrinology, Stavanger University Hospital, Stavanger, Norway; 3 Department of Medical and Clinical Psychology, Center of Research on Psychological and Somatic disorders (C*o*RPS), Tilburg University, Tilburg, the Netherlands; 4 Department of Global Public Health and Primary Care, University of Bergen, Bergen, Norway; 5 Department of Community Medicine and General Practice, Norwegian University of Science and Technology, HUNT Research Centre, Levanger, Norway; Heinrich-Heine-Universitat Dusseldorf Medizinische Fakultat, GERMANY

## Abstract

**Aim:**

To examine whether elevated anxiety and/or depressive symptoms are related to all-cause mortality in people with Type 2 diabetes, not using insulin.

**Methods:**

948 participants in the community-wide Nord-Trøndelag Health Survey conducted during 1995–97 completed the Hospital Anxiety and Depression Scale with subscales of anxiety (HADS-A) and depression (HADS-D). Elevated symptoms were defined as HADS-A or HADS-D ≥8. Participants with type 2 diabetes, not using insulin, were followed until November 21, 2012 or death. Cox regression analyses were used to estimate associations between baseline elevated anxiety symptoms, elevated depressive symptoms and mortality, adjusting for sociodemographic factors, HbA_1c_, cardiovascular disease and microvascular complications.

**Results:**

At baseline, 8% (n = 77/948) reported elevated anxiety symptoms, 9% (n = 87/948) elevated depressive symptoms and 10% (n = 93/948) reported both. After a mean follow-up of 12 years (SD 5.1, range 0–17), 541 participants (57%) had died. Participants with elevated anxiety symptoms only had a decreased mortality risk (unadjusted HR 0.66, 95% CI 0.46–0.96). Adjustment for HbA_1c_ attenuated this relation (HR 0.73, 95% CI 0.50–1.07). Those with elevated depression symptoms alone had an increased mortality risk (fully adjusted model HR 1.39, 95% CI 1.05–1.84). Having both elevated anxiety and depressive symptoms was not associated with increased mortality risk (adjusted HR 1.30, 95% CI 0.96–1.74).

**Conclusions:**

Elevated depressive symptoms were associated with excess mortality risk in people with Type 2 diabetes not using insulin. No significant association with mortality was found among people with elevated anxiety symptoms. Having both elevated anxiety and depressive symptoms was not associated with mortality. The hypothesis that elevated levels of anxiety symptoms leads to behavior that counteracts the adverse health effects of Type 2 diabetes needs further investigation.

## Introduction

People with Type 2 diabetes are more likely to experience elevated depression and/or anxiety symptoms or an anxiety disorder, than people without diabetes [1–3]. While depression affects approximately one in five individuals with Type 2 diabetes [2], generalized anxiety disorder and elevated anxiety symptoms have been reported to be present in 14% and 40%, respectively [4].

Depression was found to be associated with a higher risk of adverse diabetes outcomes and higher mortality rates [5]. Depression in diabetes is associated with suboptimal HbA_1c_ levels [6], development of vascular complications [7] and increased all-cause and cardiovascular mortality [8]. However, less is known about the health risks imposed by elevated anxiety symptoms. A meta-analysis including 11 cross-sectional studies found that anxiety disorders, but not elevated anxiety symptoms, were associated with hyperglycemia [9]. Unfortunately, most of the included studies had a small sample size (9 studies, < *n* = 125) or included clinical samples only, and only one study included only patients with Type 2 diabetes [9]. More recent cross-sectional studies examining the association between anxiety and the presence of diabetes complications have found mixed results [10–12]. Longitudinal studies focusing on the relation between anxiety and health outcomes are still lacking in people with Type 2 diabetes.

There is evidence from longitudinal cardiovascular studies that having symptoms of both anxiety and depression predicts poor health outcomes [13–15]. It is therefore relevant to test whether having not only elevated depression symptoms, but also other emotional problems such as elevated anxiety symptoms, are associated with risk of mortality Furthermore, it is unclear whether data that are collected early in the course of the disease (i.e. before insulin therapy is initiated) are associated with increased mortality risk. Therefore, we aimed to include a sample of people with less severe diabetes (i.e. not using insulin) and also report on the associations with both anxiety and depression. Moreover, the Diagnostic and Statistical Manual of Mental Disorders, Fifth Edition (DSM-5) has now added a new specification to the diagnosis of major depressive disorder: “with anxious distress”. The aim of the present study was to examine whether elevated anxiety symptoms, elevated depressive symptoms, or both, are associated with increased risk of mortality during 17 years follow up in insulin naïve people with Type 2 diabetes.

## Material and Methods

### Study sample

The Second Nord Trøndelag Health Study (HUNT2) was conducted in one large Norwegian county during 1995–1997, inviting all residents aged ≥ 20 years. The population is ethnically homogenous, with only a small percentage (3%) of people of non-Caucasian origin [16]. In total 93,898 persons were invited, 69% filled out the first general questionnaire on health behaviours, health status and demographics. A total of 1972 (3%) persons answered affirmatively on the question “Do you have or have you had diabetes?” Participants who reported to have diabetes received an additional diabetes-specific questionnaire and were invited to a follow-up visit with collection of a fasting blood sample to determine diabetes type (attendance rate: 75%). Participants were classified as having type 1 or type 2 diabetes based on a combination of three factors: (1) measures of fasting C-peptide and glutamic acid decarboxylase antibodies (anti-GAD), (2) fasting glucose levels [16]. In the diabetes-specific questionnaire, participants were asked about diabetes treatment. Those who answered that they did not use insulin were classified as respondents with insulin-naïve Type 2 diabetes. For participants who did not answer the treatment question (medication use at baseline), we retrieved information on the prescription of insulin (Anatomical Therapeutic Chemical code A10A) from the Norwegian Prescription Database (NorPD). These data showed that 126 participants who had not answered the treatment question did not use insulin. This registry includes individual prescription data from all Norwegian residents, with complete data from January 1, 2004. We linked data from HUNT2 to data from the NorPD using the 11-digit personal identification number unique to each Norwegian resident.

**Fig 1:**
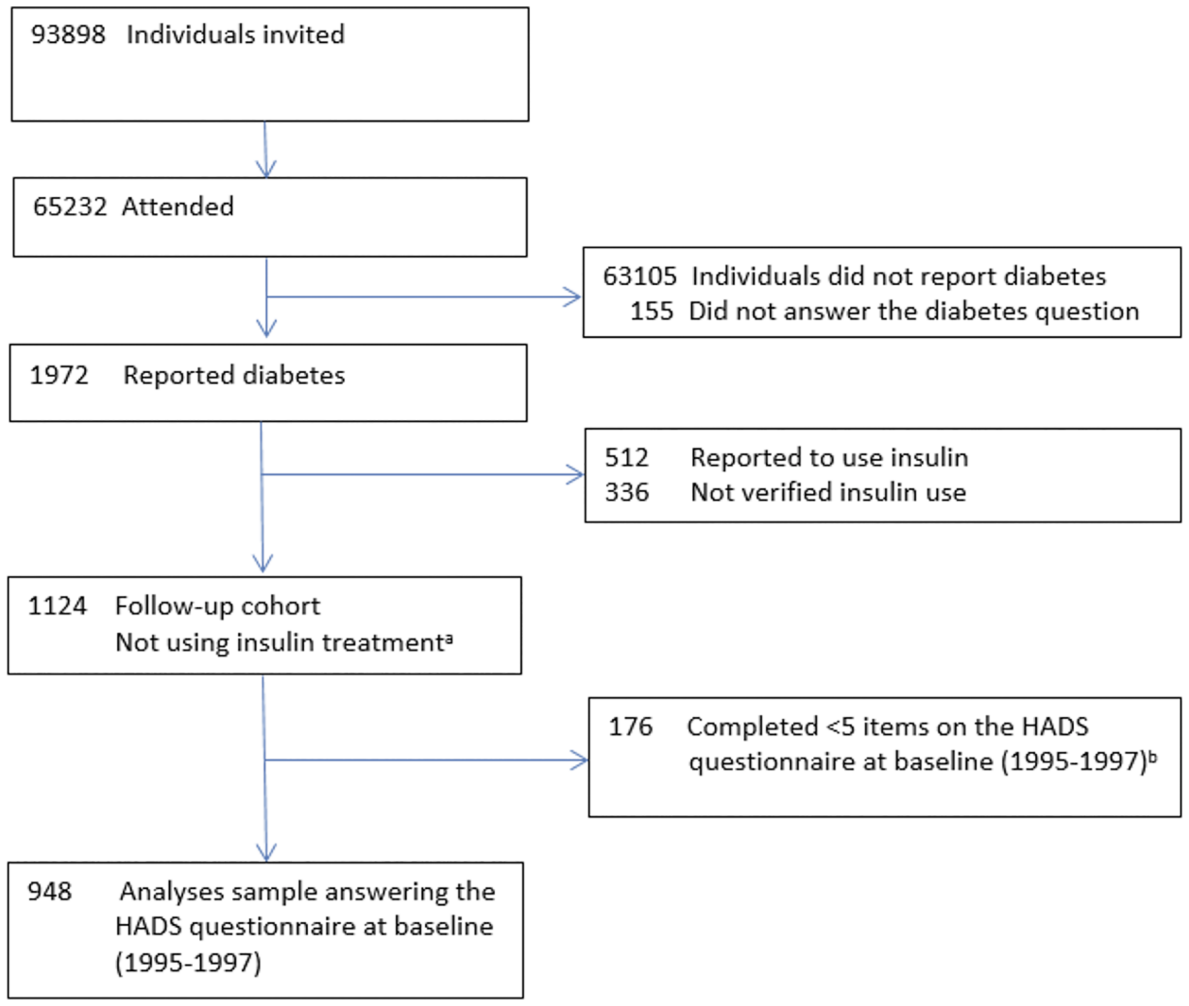
Study flowchart for the cohort recruited from HUNT2 (1995–1997). ^a^ Self-reported diabetes, verified as Type 2 diabetes and have answered not to use insulin (n = 783); self-reported diabetes, who have answered not to use insulin (n = 215); self-reported diabetes, not answering treatment question but verified as not using insulin in the Norwegian Prescription Data base (n = 126). ^b^ Completed fewer than five questions on the HADS anxiety (HADS-A) and/or depression (HADS-D) subscales.

Of 1124 participants with diabetes who were not treated with insulin, 948 completed the Hospital Anxiety and Depression Scale (HADS) with valid answers on the anxiety (HADS-A) and the HADS depression (HADS-D) subscale (responded on 5 or more questions on each subscale) (Fig 1); the analyses in the present study are based on this group.

### Assessment of anxiety and depressive symptoms

The HADS was used to assess symptoms of anxiety and depression [17]. This self-report measure was originally designed for symptom screening in hospital settings using cognitive symptoms of depression and anxiety. Somatic symptoms of depression were not used, as these can also be attributed to a somatic illness. This was done to reduce the likelihood of false-positive cases among individuals with somatic diseases [17]. The HADS anxiety and depression subscales include seven questions on general symptoms of anxiety and depression, respectively, during the past week. Each item is scored on a Likert scale ranging from 0 to 3; yielding a maximum score of 21. Higher scores indicate a higher symptom load. The HADS-A and HADS-D have previously been shown to have good psychometric properties in the HUNT2 study [18]. HADS-A and HADS-D were used as dichotomous variables with a cut-off level of ≥8 for caseness of anxiety or depression. This cut-off level has been shown to yield a sensitivity and specificity of about 0.8 on each subscale. To enhance the specificity of identifying anxiety or depression, a cut-off point of 11 is also used [19]. We imputed missing items for individuals who had responded to only five or six of the HADS-A or HADS-D questions [18]. This was done by multiplying the obtained score by 7/5 if five of the seven questions were answered and by 7/6 if six questions were answered. Such missing substitution was done for 23.5% of participants for the HADS-A scale and 8.5% for HADS-D scale. Some 15% and 10% answered fewer than five questions on HADS-A and HADS-D, respectively, and were excluded.

### Potential demographic and clinical confounders

In the general baseline questionnaire participants were asked about age, gender and their level of education. In the analyses education was categorized as a low (<10 years) or high (≥10 years) level. Living alone was categorized as yes/no. Questions related to cardiovascular disease included having a history of angina pectoris, myocardial infarction or stroke. These three conditions were combined into a composite measure of any cardiovascular disease (yes/no). The diabetes-specific questionnaire included a question about the presence of eye problems due to diabetes. Participants reported having diabetes received tubes for collecting three consecutive first-morning urine samples, which were analyzed for albumin and creatinine levels [16]. We defined microalbuminuria as an albumin-to-creatinine ratio exceeding 3.0 mg/mmol in at least two of the three urine samples [20]. Eye problems due to diabetes and microalbuminuria were combined into a composite measure of any microvascular complication (yes/no). During the follow-up visit, an EDTA (ethylenediaminetetraacetic acid) whole-blood sample was drawn for analyzing glycated hemoglobin (HbA_1c_). HbA_1c_ measurements were reported in International Federation of Clinical Chemistry units (mmol/mol) in addition to derived NGSP units (%) (http://www.ngsp.org/convert1.asp).

### Follow-up mortality data

Information on date of death was obtained from the Norwegian Population Registry. We followed participants from the baseline examination to time of death (end point) or November 21, 2012 (end of the follow-up period), whichever came first.

### Ethics

The Mid Norway Regional Committee for Medical and Health Research Ethics approved the study protocol (2011/1157), and the Norwegian Social Science Data Services approved merging the HUNT-2 data and the NorPD registry.

### Statistical analyses

All analyses were performed using IBM SPSS Statistics (version 19.0), Somers, NY, USA. A *p*-value <0.05 was considered statistically significant. Using earlier definitions of elevated symptoms of anxiety and depression [19], we created four groups based on baseline scores on the HADS-A and HADS-D subscales: (i) non-anxious and non-depressed (HADS-A <8 and HADS-D <8); (ii) anxiety symptoms only (HADS-A ≥8 and HADS-D <8); (iii) depressive symptoms only (HADS-A <8 and HADS-D ≥8); and (iv) both anxiety and depressive symptoms (HADS-A ≥8 and HADS-D ≥8). To compare baseline differences in demographic and clinical characteristics between the four groups, we used the analysis of variance (ANOVA) F test for continuous variables and the X^2^ test for categorical variables. Three planned comparisons were performed in case of statistically significant overall results, comparing the depressed-only, anxious-only and comorbid group with the non-anxious and non-depressed group as the reference group. The log-rank test was used for an overall comparison of survival curves for the four groups (visualized with Kaplan-Meier curves).

A hierarchical Cox regression analysis was then used to examine whether group status (using the group without anxiety and depressive symptoms as the reference category) was associated with increased mortality risk in an unadjusted analysis; (i) after adjustment for the demographic variables age (continuous), male sex (no or yes), level of education (high or low), and living alone (no or yes) (model 1); (ii) after adjustment for demographic variables and HbA_1c_ (model 2); and (iii) after additional adjustment for the presence of any cardiovascular disease or microvascular complications (model 3).

We compared those who completed the HADS-A and HADS-D with those who did not (fewer than 5 items), with regard to demographics and clinical variables. Furthermore, to assess the validity of the findings, we examined whether those not completing either the HADS-A or HADS-D had increased mortality risk. Cox regression analyses were used, unadjusted and with adjustments (model 3).

We conducted additional sensitivity analyses with a higher cut-off point (≥ 11) on either or both of the sub-scales to indicate moderate-severe symptomatology or clinically significant disorder [17].

## Results

At baseline in the total sample (n = 948), mean age was 67 (SD 12) years (range 20–90 years) and 49% were men. Mean HbA_1c_ level was 61mmol/mol (SD 19) (i.e. 7.7% (SD 1.7)). Cardiovascular disease and microvascular complications were present in 27% and 33% of participants, respectively. Seventy-three percent of the sample (n = 691/948) did not have elevated anxiety or depressive symptoms, 8% (n = 77) had elevated anxiety symptoms only, 9% (n = 87) had elevated depressive symptoms only, and 10% (n = 93) had both. When comparing these four groups with respect to demographic and clinical characteristics, significant overall differences were found for age, HbA_1c_ level and eye problems due to diabetes (Table 1). Planned comparisons were performed for these variables with the non-anxious and non-depressed (HADS-A <8 and HADS-D <8) as the reference group. Those with elevated anxiety symptoms only had lower HbA_1c_ levels (p<0.001); those with elevated depressive symptoms only were older (p<0.001) and those with both elevated anxiety and depressive symptoms had more eye problems due to diabetes (p<0.001) compared with individuals in the reference group (Table 1).

**Table 1:** Baseline characteristics of the study population (insulin naïve people with Type 2 diabetes), stratified by HADS-Anxiety and HADS-Depression subgroups.

Characteristics			HADS-A <8	HADS-A ≥8	HADS-A <8	HADS-A ≥8	Overall
			and	and	and	and	test
	% (n)	Number	HADS-D <8	HADS-D <8	HADS-D ≥8	HADS-D ≥8	*p*-value*
	n = 948	missing	n = 691	n = 77	n = 87	n = 93	
Sociodemographic characteristics							
Age (years), mean (SD)	67 (12)	0	67 (12)	64 (14)	71 (10)**	65 (12)	**<0.001**
Male sex, % (n)	49 (466)	0	50 (348)	39 (30)	52 (45)	46 (43)	0.25
Living alone, % (n)	39 (372)	3	41 (282)	36 (28)	31 (27)	38 (35)	0.30
Low education % (n)	65 (564)	82	64 (404)	63 (46)	70 (53)	73 (61)	0.27
Glycaemic control, mean (SD)							
HbA_1c_, % units (NGSP units)	7.7 (1.7)	43	7.8 (1.7)	7.0 (1.3)**	7.8 (1.6)	7.5 (1.4)	**<0.001**
HbA_1c_, mmol/mol (IFCCU)	61 (19)	43	62 (19)	53 (14)	62 (17)	58 (15)	
Macrovascular complications, % (n)							
Self-reported stroke ^a^	6 (55)	35	5 (33)	8 (6)	11 (9)	8 (7)	0.09
Self-reported myocardial infarction ^a^	12 (111)	21	11 (75)	11 (8)	17 (14)	15 (14)	0.35
Self-reported angina pectoris ^a^	18 (167)	26	18 (118)	18 (13)	18 (15)	23 (21)	0.67
Any cardiovascular disease ^b^	27 (250)	22	26 (173)	27 (20)	35 (30)	29 (27)	0.28
Microvascular complications, % (n)							
Microalbuminuria	23(237)	87	24 (151)	24 (17)	24 (19)	18 (15)	0.62
Eye problems due to diabetes ^a^	9 (67)	165	7 (38)	10 (6)	11 (8)	21 (15)**	**<0.001**
Any microvascular complication ^b^	33 (303)	173	31 (179)	34 (21)	37 (26)	36 (26)	0.68
HADS—A, mean score, (SD)	4.2 (3.7)		2.7 (2.1)	9.6 (2.2)	3.9 (2.0)	11.7 (2.8)	
HADS—D, mean score, (SD)	4.5 (3.4)		3.1 (2.2)	5.3 (1.8)	9.2 (1.7)	10.6 (2.4)	

^a^ Stroke, myocardial infarction, angina pectoris and eye problems due to diabetes are self-reported.

^b^ All medical comorbid conditions were combined into two composite disease measures: any cardiovascular disease (stroke, myocardial infarction and/or angina pectoris) and any microvascular complications (eye problems due to diabetes and/or microalbuminuria).

*Analysis of variance (ANOVA) F test for continuous data and X^2^ test for categorical data.

**Indicate p values < 0.001 in planned comparisons performed for variables with overall statistically significant results (p <0.05), with HADS-A <8 and HADS-D <8 as the reference group.

**Fig 2:**
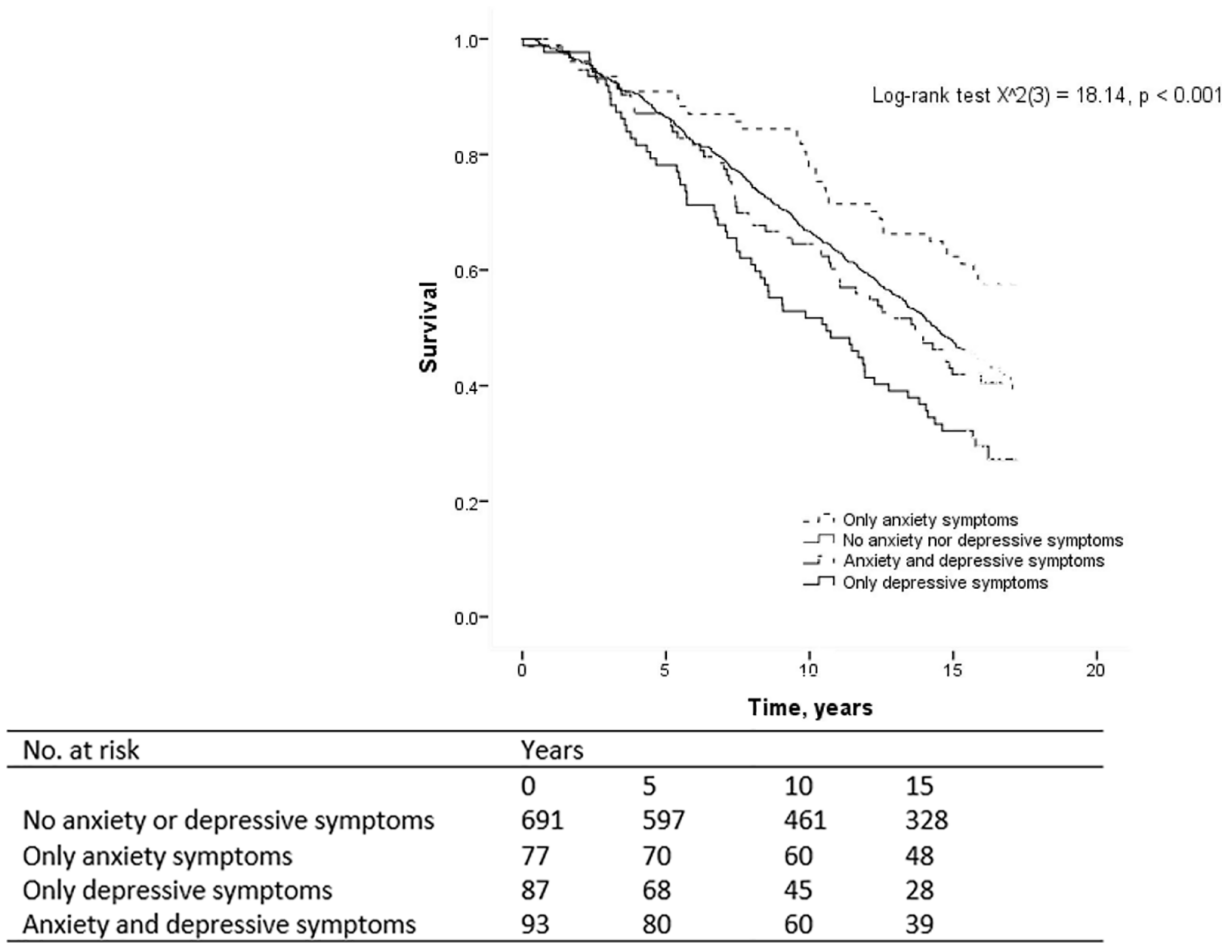
Kaplan-Meier curve for mortality comparing HADS-Anxiety and HADS-Depression subgroups.

After a mean follow-up of 12 years (SD 5.1, range 0–17), 541 people had died (57%). The survival curves for the four groups differed significantly (*p*<0.001) (Fig 2). The proportional hazards assumption was satisfied (with the correlation between the Schoenfeld residuals and ranked survival time ranging between *r* = -0.001 and -0.02 for the three comparisons, all non-significant). Crude survival analyses for these three comparisons showed that compared to the reference group of people with neither elevated anxiety nor depressive symptoms, the mortality risk was decreased for participants with elevated anxiety symptoms only (HR 0.66, 95% CI 0.46–0.96) (*p* = 0.03), and increased for participants with elevated depressive symptoms only (HR 1.59, 95% CI 1.21–2.09) (*p* = 0.001) (Table 2). Mortality risk did not differ between the reference group and participants with co-morbid elevated anxiety and depressive symptoms (HR 1.06, 95% CI 0.79–1.41) (*p* = 0.58). Adjustment for demographic variables gave similar results for all three comparisons (Model 1; Table 2). However, after further adjustment for HbA_1c_ levels, the HR for having elevated anxiety symptoms only was attenuated, changing from 0.66 to 0.73, and was no longer statistically significant (HR 0.73, 95% CI 0.50–1.07) (Model 2; *p* = 0.11). In the fully adjusted Model 3, persons with elevated depressive symptoms only had an increased mortality risk compared to the reference group (HR 1.39, 95% CI 1.05–1.84) (*p* = 0.02).

**Table 2:** Univariable and multivariable Cox proportional hazards models for the association between baseline HADS-Anxiety and HADS-Depression subgroups and all-cause mortality.

	Deaths (n)	Unadjusted HR (CI)	Model 1 HR (CI)	Model 2 HR (CI)	Model 3 HR (CI)
		n = 884 ^a^	n = 884	n = 884	n = 884
HADS- No Anxiety or depressive symptoms (scores <8)	392	Reference ^a^	Reference ^a^	Reference ^a^	Reference ^a^
Only HADS-Anxiety score ≥8	32	**0.66 (0.46–0.96)**	**0.68 (0.47–0.99)**	0.73 (0.50–1.07)	0.69 (0.47–1.10)
Only HADS-Depression score ≥8	62	**1.59 (1.21–2.09)**	**1.41 (1.07–1.89)**	**1.45 (1.10–1.92)**	**1.39 (1.05–1.84**)
HADS- Anxiety and depressive symptoms (scores ≥8)	55	1.06 (0.79–1.41)	1.30 (0.97–1.74)	**1.35 (1.01–1.81)**	1.30 (0.96–1.74)
Age (years)		**1.10 (1.09–1.11)**	**1.11 (1.09–1.12)**	**1.11 (1.09–1.12)**	**1.10 (1.09–1.12)**
Male sex		1.08 (0.91–1.28)	**1.61 (1.33–1.95)**	**1.61 (1.32–1.95)**	**1.44 (1.19–1.75)**
Low education^b^		**2.05 (1.65–2.54)**	1.11 (0.88–1.40)	1.04 (0.74–1.46)	1.11 (0.88–1.40)
Living alone		**1.50 (1.26–1.79)**	1.12 (0.93–1.35)	1.13 (0.93–1.36)	1.13 (0.94–1.37)
HbA_1c_		**1.09 (1.05–1.15)**		**1.08 (1.03–1.14)**	**1.08 (1.02–1.13)**
Any cardiovascular disease ^c^		**2.09 (1.75–2.51)**			**1.39 (1.15–1.67)**
Any microvascular complications ^b^^,^^d^		**1.93 (1.60–2.33)**			**1.35 (1.11–1.65)**

Cox regression analysis was used to examine whether group status was associated with increased mortality risk in an unadjusted analysis; after adjustment for demographics (model 1); after adjustment for demographics and HbA_1c_ (model 2); and after adjustment for demographics, HbA_1c_, and the presence of any cardiovascular disease or microvascular complication (model 3). Data are hazard ratios (HR) with 95% CI.

^a^ Reference category.

^b^ Missing values entered as separate category.

^c^ Self-reported stroke, myocardial infarction and/or angina pectoris, as reported at baseline.

^d^ Albumin/creatinine ratio >3.0 mg/mmol in at least two of the three urine samples was used to define microalbuminuria. Eye problems caused by diabetes and/or microalbuminuria were combined into a composite measure of any microvascular complication.

Some individuals did not complete either the HADS-A (15%) or HADS-D (10%) subscale, or neither. Those not completing either scale were older, more likely to be women, to have lower education, more cardiovascular disease and microvascular complications. Those not completing the HADS-D were more likely to live alone. Non-responders on HADS-A had an increased mortality risk compared to responders in the unadjusted analysis (HR 1.92, 95% CI 1.59–2.32) (p<0.001) and in the fully adjusted Model 3 (HR 1.42, 95% CI 1.42–1.76) (p = 0.02). Non-responders on HADS-D had an increased mortality risk compared to responders in the unadjusted analysis (HR 2.28, 95% CI 1.76–2.78) (p<0.001) and in the fully adjusted Model 3 (HR 1.63, 95% CI 1.28–2.10) (p<0.001).

The results from the sensitivity analysis with groups defined by HADS ≥ 11 were analogous to our results for HADS ≥ 8. Compared to the reference group (n = 790), we observed an increased mortality rate among participants with elevated depression symptoms only (n = 30) (HR 1.36, 95% CI 0.88–2.11) (model 3). Probably due to a lack of power, this association was not significant. The mortality rate did also not differ statistically between the reference group and participants with elevated anxiety symptoms only (n = 39) (HR 0.85, 95% CI 0.52–1.39) and those with co-morbid elevated anxiety and depressive symptoms (n = 25) (HR 1.60, 95% CI 0.97–2.64).

## Discussion

In this 17-year follow-up study of 948 people with Type 2 diabetes not using insulin, baseline depression only (defined as a high level of depressive symptoms, without a co-morbid high level of anxiety symptoms) was associated with a 39% increased mortality risk after adjustment for demographics, HbA_1c_, and comorbidities. In contrast, anxiety only (defined as a high level of anxiety symptoms, without a co-morbid high level of depressive symptoms) was associated with a 34% decreased mortality risk in unadjusted analysis. However, this association was attenuated to a 27% and no longer statistically significant reduced risk after adjustment for differences in demographics and baseline HbA_1c_. Having both elevated anxiety and depression symptoms was not associated with increased or decreased mortality risk.

Our study is the first to investigate the associations between increased baseline symptom levels of anxiety, depression (and their co-morbidity) and subsequent mortality in a large community-based population of people with insulin naïve Type 2 diabetes. The finding that particularly depression is associated with increased mortality rates is in line with results of a meta-analysis which showed that depression was associated with a 46% higher mortality rate in persons with diabetes [8]. Cuijpers et al [21] found comparable pooled risk ratios for depression with 52% higher mortality rates both in general communities and in clinical samples [21]. General communities included relatively healthy samples and specific clinic patient samples with heart disease, cancer and kidney disease [21]. However, these studies did not examine whether the relation between depression and mortality was less pronounced in those with co-morbid anxiety.

Longitudinal studies focusing on the relation between anxiety and health outcomes are lacking in people with Type 2 diabetes. In this follow-up study, reporting elevated anxiety symptoms only was associated with a decreased mortality risk, partly explained by more optimal levels of HbA_1c_ in those reporting elevated anxiety symptoms. Two recent cross-sectional studies among people with Type 1 diabetes investigated the association between anxiety and HbA_1c_ levels [22, 23]. The results in the study of Strandberg [22] showed no significant association between levels of anxiety and HbA_1c_. However, results in the study of Anderbro [23] showed a strong relationship between fear of hypoglycemia, other diabetes-related fears, and non-diabetes-related types of anxiety (measured by HADS). Moreover, elevated anxiety symptoms were associated with a more favorable HbA_1c_. Similarly in our study, participants with elevated anxiety symptoms only had lower HbA_1c_ levels compared with individuals with no elevated level of anxiety or depressive symptoms. In contrast to participants with Type 1 diabetes in the study of Anderbro [23] participants in our study did not use insulin at baseline. Even though pathways may differ between people with Type 1 and Type 2 diabetes (e.g. different treatment therapies and age differences at onset), the prevalence of elevated anxiety symptoms is similar in people with Type 1 and Type 2 diabetes [4]. One explanation for our finding that having more anxiety symptoms was associated with a more favorable HbA_1c_ may be that anxious persons with diabetes are more cautious about their blood glucose level and worry about diabetes-related complications such as macro- and microvascular complications. Also, anxious people with insulin-naïve Type 2 diabetes more often fear hypoglycemia, insulin injections or blood glucose self-testing, a fear which has shown to be a barrier to insulin treatment in previous studies [24, 25]. Previously, we found that elevated levels of anxiety symptoms were associated with a later initiation of insulin, which appeared to be partly explained by better glycemic control in the anxious group, while elevated depressive symptoms were not. Persons with both elevated levels of anxiety and depression were also less likely to start insulin therapy [26]. In a longitudinal Dutch study, depression was not associated with initiation of insulin therapy in people with type 2 diabetes [27]. Other studies have found that insulin treatment is associated with increased mortality rates [28] and it is possible that during this follow-up HbA_1c_ levels increased and people subsequently started with insulin treatment which is associated with a worse prognosis or more advanced disease. Treatment adherence may be a mediator into the association between elevated anxiety symptoms and lower HbA_1c_ levels. In this case, elevated anxiety scores might be a marker for a dedicated attitude towards minimizing health risks. It could be speculated that better treatment adherence is more often adopted as coping style in anxious people compared to other participants with less anxiety [29]. Treatment adherence may therefore explain the better glycemic outcome and subsequent better survival rates.

Furthermore, we found that having elevated symptoms of both anxiety and depression was not associated with an increased mortality rate. Lou et al. [30] found different results in a large study among 7,787 patients with chronic obstructive lung disease. In their study, depression and anxiety had an additive effect on mortality rates. In the WISE study [31], however, depression scores were significant predictors of CVD events among women with low anxiety scores (hazard ratio 2.3), but not among women with higher levels of anxiety (hazard ratio 0.99). This suggests a health-protective role for anxiety, with anxiety counteracting the adverse health effects of depression. Similarly, in the present study, decreased mortality was observed among people with elevated anxiety symptoms. However, this was in part explained by lower HbA_1c_ levels.

Our study has several strengths. We have studied anxiety and depression as risk factors early in the course of the disease by including the large, relatively homogenous sample of insulin naïve people with Type 2 diabetes who were followed for a relatively long period with mortality as the endpoint. At the same time, this is also a limitation, as we could not compare our sample to people using insulin. However, it was our *a priori* aim to test depressive symptoms as a risk factor for mortality, with depression being measured in an early, relatively uncomplicated stage of diabetes. Depression may be a consequence of complications of diabetes, which again is associated with increased mortality risk, but this was not the focus of the paper. We therefore requested data from participants with type 2 diabetes who were insulin-naïve at baseline and data from participants who using insulin at baseline are thus not available to us. Hence, we do not have the possibility to re-run the analysis including those using insulin. It is a strength is that the self-reported assessments used do not include somatic symptoms of depression or anxiety thus diminishing the effect of somatic illness such as e.g. diabetes. On the other hand, assessment of depression and anxiety symptoms was by means of a self-report measure, while a psychiatric diagnostic interview is regarded as the gold standard for assessing mood and anxiety disorders. We are aware of the fact that the validity of the HADS questionnaire has been the subject of a recent debate. Coyne et al [32] stated that the HADS has no reliable, generalisable latent structure and questioned its continued use as a screening and assessment instrument for major depression and anxiety. However, Norton et al [33] stated in their response that these limitations are not unique for the HADS, other depression screening tools suffer from the same problems. In addition, other study limitations should be discussed. First, we used self-reported diabetes, which may induce selection bias, since people with anxiety may be less likely to have (mild) undetected diabetes. Second, we have no information on possible changes in health behaviours and treatment of diabetes during the follow-up period. We have only measurements of anxiety and depressive symptoms at baseline and therefore we do not know the trajectories in the follow-up. However, the study of Nefs [34] has shown that a history of depression is the main risk factor for depression later in life. Once present, depression often becomes a chronic/recurrent condition in this group. Third, non-responders on the HADS questionnaire reported lower education, more self-reported cardiovascular disease and self-reported eye problems due to diabetes and had increased mortality risk, corresponding to results from other studies [35]. Fourth, analyses might be underpowered due to due to small numbers of participants in the groups (anxiety symptoms only (HADS-A ≥8 and HADS-D <8); depressive symptoms only (HADS-A <8 and HADS-D >8); and both anxiety and depressive symptoms (HADS-A ≥8 and HADS-D ≥8)). Finally, we could not take into account neuropathy in the analysis, as we do not have these clinical data.

In sum, in a cohort of insulin-naïve participants with Type 2 diabetes followed over a period of 17 years, an elevated level of depressive symptoms (without elevated anxiety symptoms) was associated with a 39% increased mortality risk. A similar association was not observed among people with elevated anxiety symptoms only. While decreased mortality was observed in this group, it was in part explained by lower HbA_1c_ levels. This may be interpreted as lending weight to a hypothesis of elevated levels of anxiety symptoms leading to behavior that counteract the adverse health effects of Type 2 diabetes. This hypothesis needs further investigations in future research.

## References

[1] Smith KJ, Beland M, Clyde M, Gariepy G, Page V, Badawi G, et al (2013) Association of diabetes with anxiety: a systematic review and meta-analysis. J Psychosom Res 74: 89–99. 10.1016/j.jpsychores.2012.11.013 23332522

[2] Ali S, Stone MA, Peters JL, Davies MJ, Khunti K (2006) The prevalence of co-morbid depression in adults with Type 2 diabetes: a systematic review and meta-analysis. Diabet Med 23: 1165–73. 17054590 10.1111/j.1464-5491.2006.01943.x

[3] Nouwen A, Winkley K, Twisk J, Lloyd CE, Peyrot M, Ismail K, et al (2010) Type 2 diabetes mellitus as a risk factor for the onset of depression: a systematic review and meta-analysis. Diabetologia 53: 2480–6. 20711716 10.1007/s00125-010-1874-x PMC2974923

[4] Grigsby AB, Anderson RJ, Freedland KE, Clouse RE, Lustman PJ (2002) Prevalence of anxiety in adults with diabetes: a systematic review. J Psychosom Res 53: 1053–60. 12479986 10.1016/s0022-3999(02)00417-8

[5] Pouwer F, Nefs G, Nouwen A (2013) Adverse effects of depression on glycemic control and health outcomes in people with diabetes: a review. Endocrinol Metab Clin North Am 42: 529–44. 10.1016/j.ecl.2013.05.002 24011885

[6] Lustman PJ, Anderson RJ, Freedland KE, de Groot M, Carney RM, Clouse RE (2000) Depression and poor glycemic control: a meta-analytic review of the literature. Diabetes Care 23: 934–42. 10895843 10.2337/diacare.23.7.934

[7] Lin EH, Rutter CM, Katon W, Heckbert SR, Ciechanowski P, Oliver MM, et al (2010) Depression and advanced complications of diabetes: a prospective cohort study. Diabetes Care 33: 264–9. 10.2337/dc09-1068 19933989 PMC2809260

[8] Dooren FEv, Nefs G, Schram MT, Verhey FR, Denollet J, Pouwer F (2013) Depression and risk of mortality in people with diabetes mellitus: a systematic review and meta-analysis. PLoS One 8: e57058 10.1371/journal.pone.0057058 23472075 PMC3589463

[9] Anderson RJ, de Groot M, Grigsby AB, McGill JB, Freedland KE, Clouse RE, et al (2002) Anxiety and poor glycemic control: A meta-analytic review of the literature. Int J Psychiatry Med 32: 235–47. 12489699 10.2190/KLGD-4H8D-4RYL-TWQ8

[10] Hermanns N, Kulzer B, Krichbaum M, Kubiak T, Haak T (2005) Affective and anxiety disorders in a German sample of diabetic patients: prevalence, comorbidity and risk factors. Diabet Med 22: 293–300. 15717877 10.1111/j.1464-5491.2005.01414.x

[11] Masmoudi J, Damak R, Zoari H, Ouali U, Mechri A, Zouari N, et al (2013) Prevalence and Impact of Anxiety and Depression on Type 2 Diabetes in Tunisian Patients over Sixty Years Old. Depression Research and Treatment 2013:341782. 23853718 10.1155/2013/341782 PMC3703715

[12] Tovilla-Zarate C, Juarez-Rojop I, Peralta Jimenez Y, Jimenez MA, Vazquez S, Bermudez-Ocana D, et al (2012) Prevalence of anxiety and depression among outpatients with type 2 diabetes in the Mexican population. PLoS ONE 7: e36887 10.1371/journal.pone.0036887 22629339 PMC3356343

[13] Doering LV, Moser DK, Riegel B, McKinley S, Davidson P, Baker H, et al (2010) Persistent comorbid symptoms of depression and anxiety predict mortality in heart disease. Int J Cardiol 145: 188–92. 10.1016/j.ijcard.2009.05.025 19493579 PMC2998562

[14] Watkins LL, Koch GG, Sherwood A, Blumenthal JA, Davidson JR, O'Connor C, et al (2013) Association of anxiety and depression with all-cause mortality in individuals with coronary heart disease. J Am Heart Assoc 2: e000068 10.1161/JAHA.112.000068 23537805 PMC3647264

[15] Park JH, Tahk SJ, Bae SH (2015) Depression and Anxiety as Predictors of Recurrent Cardiac Events 12 Months After Percutaneous Coronary Interventions. J Cardiovasc Nurs 30:351–9 2014. 10.1097/JCN.0000000000000143 24763357

[16] Holmen J, Midthjell K, Kruger Ø, Langhammer A, Holmen TL, Bratberg GH, et al (2003) The Nord-Trøndelag Health Study 1995–97 (HUNT 2): Objectives, contents, methods and participation. Norsk Epidemiologi 13: 19–32.

[17] Zigmond AS, Snaith RP (1983) The Hospital Anxiety and Depression Scale. Acta Psychiatr Scand 67: 361–70. 6880820 10.1111/j.1600-0447.1983.tb09716.x

[18] Mykletun A, Stordal E, Dahl AA (2001) Hospital Anxiety and Depression (HAD) scale: factor structure, item analyses and internal consistency in a large population. Br J Psychiatry 179: 540–4. 11731359 10.1192/bjp.179.6.540

[19] Bjelland I, Dahl AA, Haug TT, Neckelmann D (2002) The validity of the Hospital Anxiety and Depression Scale. An updated literature review. J Psychosom Res 52: 69–77. 11832252 10.1016/s0022-3999(01)00296-3

[20] American Diabetes Association (2009) Standards of medical care in diabetes. Diabetes Care 32 Suppl 1: S13–61. 10.2337/dc09-S013 19118286 PMC2613589

[21] Cuijpers P, Vogelzangs N, Twisk J, Kleiboer A, Li J, Penninx BW (2014) Comprehensive meta-analysis of excess mortality in depression in the general community versus patients with specific illnesses. Am J Psychiatry 171: 453–62. 10.1176/appi.ajp.2013.13030325 24434956

[22] Strandberg R, Graue M, Wentzel-Larse nT, Peyrot M, Rokne B (2014) Relationships of diabetes-specific emotional distress, depression, anxiety, and overall well-being with HbA1c in adult persons with type 1 diabetes. J Psychosom Res 77: 174–9. 10.1016/j.jpsychores.2014.06.015 25149027

[23] Anderbro T, Gonder-Frederick L, Bolinder J, Lins P-E, Wredling R, Moberg E, et al (2015) Fear of hypoglycemia: relationship to hypoglycemic risk and psychological factors. Acta Diabetol 52: 581–9. 10.1007/s00592-014-0694-8 25528005

[24] Nam S, Chesla C, Stotts NA, Kroon L, Janson SL (2010) Factors associated with psychological insulin resistance in individuals with type 2 diabetes. Diabetes Care 33: 1747–9. 10.2337/dc10-0099 20435797 PMC2909055

[25] Polonsky WH, Fisher L, Guzman S, Villa-Caballero L, Edelman SV (2005) Psychological insulin resistance in patients with type 2 diabetes: the scope of the problem. Diabetes Care 28: 2543–5. 16186296 10.2337/diacare.28.10.2543

[26] Iversen MM, Nefs G, Tell GS, Espehaug B, Midthjell K, Graue M, et al (2015) Anxiety, depression and timing of insulin treatment among people with type 2 diabetes: Nine-year follow-up of the Nord-Trøndelag Health Study, Norway. J Psychosom Res 79: 309–15. 10.1016/j.jpsychores.2015.07.004 26208403

[27] Nefs G, Pop VJ, Denollet J, Pouwer F (2013) The longitudinal association between depressive symptoms and initiation of insulin therapy in people with type 2 diabetes in primary care. PLoS ONE 8: e78865 10.1371/journal.pone.0078865 24223860 PMC3815321

[28] Saleh N, Petursson P, Lagerqvist B, Skuladottir H, Svensson A, Eliasson B, et al (2012) Long-term mortality in patients with type 2 diabetes undergoing coronary angiography: the impact of glucose-lowering treatment. Diabetologia 55: 2109–17. 10.1007/s00125-012-2565-6 22566103

[29] Vallis M, Jones A, Pouwer F. (2014) Managing hypoglycemia in diabetes may be more fear management than glucose management: a practical guide for diabetes care providers. Curr Diabetes Rev 10:364–70. 25394991 10.2174/1573399810666141113115026

[30] Lou P, Zhu Y, Chen P, Zhang P, Yu J, Wang Y, et al (2014) Interaction of depressive and anxiety symptoms on the mortality of patients with COPD: a preliminary study. Copd 11: 444–50. 10.3109/15412555.2013.822856 25010754

[31] Rutledge T, Linke SE, Krantz DS, Johnson BD, Bittner V, Eastwood JA, et al (2009) Comorbid depression and anxiety symptoms as predictors of cardiovascular events: results from the NHLBI-sponsored Women's Ischemia Syndrome Evaluation (WISE) study. Psychosom Med 71: 958–64. 10.1097/PSY.0b013e3181bd6062 19834049 PMC2783707

[32] Coyne JC, Van Sonderen E. No further research needed: abandoning the Hospital and Anxiety Depression Scale (HADS). Journal of Psychosomatic Research 2012;72:173–4. 10.1016/j.jpsychores.2011.12.003 22325694

[33] Norton S, Sacker A, Done J. Further research needed: a comment on Coyne and van Sonderen's call to abandon the Hospital Anxiety and Depression Scale. Journal of Psychosomatic Research 2012;73:75–6. 10.1016/j.jpsychores.2012.04.005 22691565

[34] Nefs G, Pouwer F, Denollet J, Pop V. (2012) The course of depressive symptoms in primary care patients with type 2 diabetes: results from the Diabetes, Depression, Type D Personality Zuidoost-Brabant (DiaDDZoB) Study. Diabetologia. 55:608–16. 10.1007/s00125-011-2411-2 22198261 PMC3268983

[35] Drivsholm T, Eplov LF, Davidsen M, Jørgensen T, Ibsen H, Hollnagel H, Borch-Johnsen K. (2006) Representativeness in population-based studies: a detailed description of non-response in a Danish cohort study. Scand J Public Health 34:623–31. 17132596 10.1080/14034940600607616

